# 3-Phenyl-1*H*-1,2,4-triazol-5-amine–5-phenyl-1*H*-1,2,4-triazol-3-amine (1/1)

**DOI:** 10.1107/S1600536808042165

**Published:** 2008-12-17

**Authors:** Anton V. Dolzhenko, Geok Kheng Tan, Lip Lin Koh, Anna V. Dolzhenko, Wai Keung Chui

**Affiliations:** a Department of Pharmacy, Faculty of Science, National University of Singapore, 18 Science Drive 4, Singapore 117543, Singapore; b Department of Chemistry, Faculty of Science, National University of Singapore, 3 Science Drive 3, Singapore 117543, Singapore

## Abstract

In the title compound, C_8_H_8_N_4_·C_8_H_8_N_4_, two tautomers, *viz*. 3-phenyl-1,2,4-triazol-5-amine and 5-phenyl-1,2,4-triazol-3-amine, are crystallized together in equal amounts. The 3-phenyl-1,2,4-triazol-5-amine mol­ecule is essentially planar; the phenyl ring makes a dihedral angle of 2.3 (2)° with the mean plane of the 1,2,4-triazole ring. In the 5-phenyl-1,2,4-triazol-3-amine tautomer, the mean planes of the phenyl and 1,2,4-triazole rings form a dihedral angle of 30.8 (2)°. The π-electron delocalization of the amino group with the 1,2,4-triazole nucleus in the 3-phenyl-1,2,4-triazol-5-amine mol­ecule is more extensive than that in the 5-phenyl-1,2,4-triazol-3-amine tautomer. The mol­ecules are linked into a two-dimensional network parallel to (100) by N—H⋯N hydrogen bonds.

## Related literature

For a summary of structural data for 1,2,4-triazoles, see: Buzykin *et al.* (2006 ▶). For the crystal structure of 3-pyridin-2-yl-1,2,4-triazol-5-amine, see: Dolzhenko *et al.* (2009 ▶). For the use of 1,2,4-triazol-5-amines as building blocks in the synthesis of fused heterocyclic systems, see: Dolzhenko *et al.* (2006 ▶, 2007*a* ▶,*b* ▶); Fischer (2007 ▶).
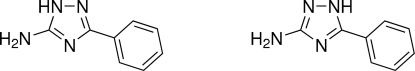


## Experimental

### 

#### Crystal data

C_8_H_8_N_4_·C_8_H_8_N_4_
*M* *_r_* = 320.36
Monoclinic, 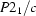

*a* = 17.817 (2) Å
*b* = 5.0398 (6) Å
*c* = 18.637 (2) Å
β = 113.573 (4)°
*V* = 1533.9 (3) Å^3^
*Z* = 4
Mo *K*α radiation
μ = 0.09 mm^−1^
*T* = 223 (2) K
0.60 × 0.10 × 0.06 mm

#### Data collection

Bruker SMART APEX CCD diffractometer
Absorption correction: multi-scan (*SADABS*; Sheldrick, 2001 ▶) *T*_min_ = 0.947, *T*_max_ = 0.995
10288 measured reflections
3523 independent reflections
2394 reflections with *I* > 2σ(*I*)
*R*_int_ = 0.063

#### Refinement

*R*[*F* ^2^ > 2σ(*F* ^2^)] = 0.067
*wR*(*F* ^2^) = 0.168
*S* = 0.99
3523 reflections
241 parameters
H atoms treated by a mixture of independent and constrained refinement
Δρ_max_ = 0.43 e Å^−3^
Δρ_min_ = −0.24 e Å^−3^

### 

Data collection: *SMART* (Bruker, 2001 ▶); cell refinement: *SAINT* (Bruker, 2001 ▶); data reduction: *SAINT*; program(s) used to solve structure: *SHELXS97* (Sheldrick, 2008 ▶); program(s) used to refine structure: *SHELXS97* (Sheldrick, 2008 ▶); molecular graphics: *SHELXTL* (Sheldrick, 2008 ▶); software used to prepare material for publication: *SHELXTL*.

## Supplementary Material

Crystal structure: contains datablocks I, global. DOI: 10.1107/S1600536808042165/ci2720sup1.cif

Structure factors: contains datablocks I. DOI: 10.1107/S1600536808042165/ci2720Isup2.hkl

Additional supplementary materials:  crystallographic information; 3D view; checkCIF report

## Figures and Tables

**Table 1:** Hydrogen-bond geometry (Å, °)

*D*—H⋯*A*	*D*—H	H⋯*A*	*D*⋯*A*	*D*—H⋯*A*
N3—H3*N*⋯N2^i^	0.89 (3)	2.08 (3)	2.966 (3)	175 (3)
N4—H4*A*⋯N1^ii^	0.92 (3)	2.09 (3)	3.011 (3)	173 (3)
N4—H4*B*⋯N8^i^	0.85 (3)	2.25 (3)	3.091 (3)	170 (3)
N6—H6*N*⋯N5^iii^	0.87 (4)	2.04 (4)	2.879 (3)	159 (3)
N8—H8*A*⋯N2	0.81 (3)	2.41 (3)	3.206 (3)	168 (3)
N8—H8*B*⋯N7^iv^	0.94 (4)	2.19 (4)	3.115 (3)	169 (3)

Symmetry codes: (i) 
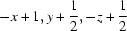
; (ii) 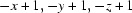; (iii) 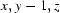; (iv) 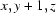.

**Fig. 1.**
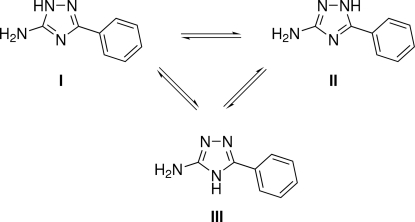
Possible tautomers of 3(5)-phenyl-1,2,4-triazol-5(3)-amine.

**Fig. 2.**
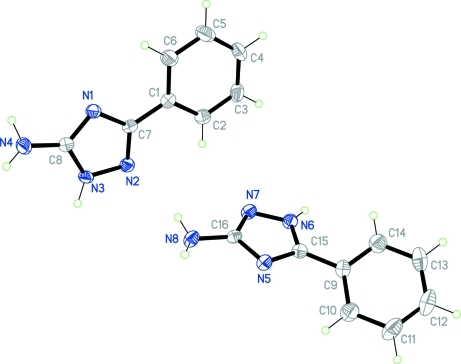
The molecular structure of 3(5)-phenyl-1,2,4-triazol-5(3)-amine with the atomic numbering scheme. Displacement ellipsoids are drawn at the 50% probability level.

**Fig. 3.**
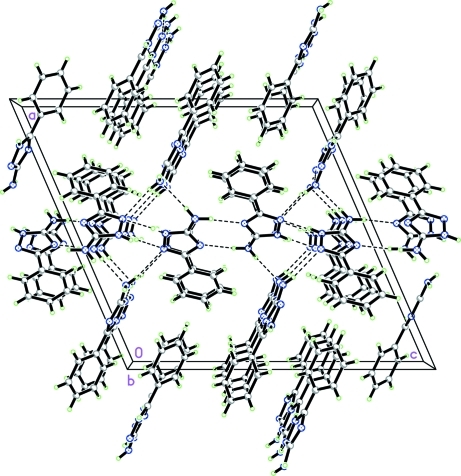
Molecular packing in the crystal, viewed along the b axis. Hydrogen bonds are shown as dashed lines.

## supplementary crystallographic information

### Comment

1,2,4-Triazol-5-amines have been used as building blocks for the synthesis of fused heterocyclic systems, *e.g.* 1,2,4-triazolo[1,5-*a*]pyrimidines (Fischer, 2007) and 1,2,4-triazolo[1,5-*a*][1,3,5]triazines (Dolzhenko *et al.*, 2006). Herein, we report the structural study of 3(5)-phenyl-1,2,4-triazol-5(3)-amine, which was used as a synthon in our previous works (Dolzhenko *et al.*, 2007*a*,b).

Due to annular tautomerism in 1,2,4-triazole ring, there is a theoretical possibility of three tautomeric forms, namely 3-phenyl-1,2,4-triazol-5-amine (I), 5-phenyl-1,2,4-triazol-3-amine (II), and 5-phenyl-4*H*-1,2,4-triazol-3-amine (III) (Fig.1).

Usually, tautomerizable 1,2,4-triazoles with nonequivalent substituents at positions 3 and 5 crystallize as a tautomer bearing at position 5 substituent with relatively more pronounced electronodonor properties (Buzykin *et al.*, 2006). Considering significant difference in electronic properties of phenyl and amino group, the crystal would be assembled from the molecules of tautomer I analogously to the reported 3-pyridin-2-yl-1,2,4-triazol-5-amine (Dolzhenko *et al.*, 2009). Surprisingly, two tautomeric forms I and II were found crystallized together in the crystal. To the best of our knowledge, this is the first example of existence in crystal of unequally 3,5-disubstituted tautomerizable 1,2,4-triazole tautomeric form with electronodonor group located at position 3.

The geometry of the tautomer I molecule is essentially planar (Fig.2). The amino group is involved in π-electron delocalization with the 1,2,4-triazole nucleus. It is almost planar with small deviation 0.06 (2) Å of the nitrogen atom from the C8/H4A/H4B plane. The length of the C8—N4 bond is 1.337 (3) Å. The π-electron delocalization of the amino group of II with the 1,2,4-triazole nucleus is significantly lower. The nitrogen atom (N8) of the amino group adopts a pyramidal configuration with 0.21 (2) Å deviation of the nitrogen atom from the C16/H8A/H8B plane. The C16—N8 bond [1.372 (3) Å] is also longer. The phenyl ring of I makes a small dihedral angle of 2.3 (2)° with the mean plane of the 1,2,4-triazole ring. The molecule of tautomer II loses this planarity. The mean planes of the phenyl and 1,2,4-triazole rings of II form a dihedral angle of 30.8 (2)°.

The molecules are linked into a two-dimensional network parallel to the (100) by N—H···N hydrogen bonds (Table 1 and Fig.3).

### Experimental

(5)-Phenyl-1,2,4-triazol-5(3)-amine was prepared according to Dolzhenko *et al.* (2007*a*,b). The crystals suitable for crystallographic analysis were grown by recrystallization from ethanol.

### Refinement

N-bound H-atoms were located in a difference map and refined freely. C-bound H atoms were positioned geometrically (C-H = 0.94 Å) and were constrained in a riding motion approximation with *U*_iso_(H) = 1.2*U*_eq_(C).

### Crystal data

**Table:**

C_8_H_8_N_4_·C_8_H_8_N_4_	*F*(000) = 672
*M**_r_* = 320.36	*D*_x_ = 1.387 Mg m^−^^3^
Monoclinic, *P*2_1_/*c*	Melting point: 460 K
Hall symbol: -P 2ybc	Mo *K*α radiation, λ = 0.71073 Å
*a* = 17.817 (2) Å	Cell parameters from 1028 reflections
*b* = 5.0398 (6) Å	θ = 2.4–22.6°
*c* = 18.637 (2) Å	µ = 0.09 mm^−^^1^
β = 113.573 (4)°	*T* = 223 K
*V* = 1533.9 (3) Å^3^	Rod, colourless
*Z* = 4	0.60 × 0.10 × 0.06 mm

### Data collection

**Table:**

Bruker SMART APEX CCD diffractometer	3523 independent reflections
Radiation source: fine-focus sealed tube	2394 reflections with *I* > 2σ(*I*)
graphite	*R*_int_ = 0.063
φ and ω scans	θ_max_ = 27.5°, θ_min_ = 2.2°
Absorption correction: multi-scan (*SADABS*; Sheldrick, 2001)	*h* = −17→23
*T*_min_ = 0.947, *T*_max_ = 0.995	*k* = −6→6
10288 measured reflections	*l* = −24→20

### Refinement

**Table:**

Refinement on *F*^2^	Primary atom site location: structure-invariant direct methods
Least-squares matrix: full	Secondary atom site location: difference Fourier map
*R*[*F*^2^ > 2σ(*F*^2^)] = 0.067	Hydrogen site location: inferred from neighbouring sites
*wR*(*F*^2^) = 0.168	H atoms treated by a mixture of independent and constrained refinement
*S* = 0.99	*w* = 1/[σ^2^(*F*_o_^2^) + (0.0813*P*)^2^ + 0.5041*P*] where *P* = (*F*_o_^2^ + 2*F*_c_^2^)/3
3523 reflections	(Δ/σ)_max_ = 0.001
241 parameters	Δρ_max_ = 0.43 e Å^−^^3^
0 restraints	Δρ_min_ = −0.24 e Å^−^^3^

### Special details

**Table:**

Geometry. All esds (except the esd in the dihedral angle between two l.s. planes) are estimated using the full covariance matrix. The cell esds are taken into account individually in the estimation of esds in distances, angles and torsion angles; correlations between esds in cell parameters are only used when they are defined by crystal symmetry. An approximate (isotropic) treatment of cell esds is used for estimating esds involving l.s. planes.
Refinement. Refinement of *F*^2^ against ALL reflections. The weighted *R*-factor *wR* and goodness of fit *S* are based on *F*^2^, conventional *R*-factors *R* are based on *F*, with *F* set to zero for negative *F*^2^. The threshold expression of *F*^2^ > σ(*F*^2^) is used only for calculating *R*-factors(gt) *etc*. and is not relevant to the choice of reflections for refinement. *R*-factors based on *F*^2^ are statistically about twice as large as those based on *F*, and *R*- factors based on ALL data will be even larger.

### Fractional atomic coordinates and isotropic or equivalent isotropic displacement parameters (Å^2^)

**Table:**

	*x*	*y*	*z*	*U*_iso_*/*U*_eq_	
N1	0.45958 (12)	0.2679 (4)	0.41243 (11)	0.0227 (5)	
N2	0.44655 (12)	0.1498 (4)	0.29112 (11)	0.0228 (5)	
N3	0.50228 (12)	0.3557 (4)	0.31965 (12)	0.0232 (5)	
H3N	0.5206 (17)	0.441 (6)	0.2883 (17)	0.034 (8)*	
N4	0.55437 (15)	0.6210 (5)	0.43462 (14)	0.0306 (5)	
H4A	0.5543 (18)	0.650 (6)	0.483 (2)	0.047 (9)*	
H4B	0.5900 (18)	0.694 (6)	0.4217 (17)	0.038 (8)*	
C1	0.36387 (14)	−0.0976 (5)	0.34673 (13)	0.0221 (5)	
C2	0.32601 (15)	−0.2542 (5)	0.28099 (14)	0.0275 (6)	
H2	0.3384	−0.2297	0.2369	0.033*	
C3	0.26999 (16)	−0.4464 (5)	0.27973 (16)	0.0326 (6)	
H3	0.2450	−0.5525	0.2349	0.039*	
C4	0.25056 (16)	−0.4837 (5)	0.34337 (16)	0.0317 (6)	
H4	0.2119	−0.6128	0.3420	0.038*	
C5	0.28814 (18)	−0.3303 (6)	0.40898 (17)	0.0405 (7)	
H5	0.2754	−0.3557	0.4528	0.049*	
C6	0.34456 (17)	−0.1385 (6)	0.41101 (16)	0.0352 (7)	
H6	0.3700	−0.0352	0.4563	0.042*	
C7	0.42378 (14)	0.1068 (5)	0.34941 (13)	0.0205 (5)	
C8	0.50768 (14)	0.4241 (5)	0.39146 (13)	0.0223 (5)	
N5	0.18197 (12)	0.2602 (4)	0.01779 (11)	0.0225 (5)	
N6	0.17613 (13)	−0.1706 (4)	0.02735 (12)	0.0241 (5)	
H6N	0.165 (2)	−0.339 (7)	0.0188 (19)	0.057 (10)*	
N7	0.25003 (12)	−0.1005 (4)	0.08543 (11)	0.0247 (5)	
N8	0.31434 (13)	0.3193 (5)	0.12197 (13)	0.0257 (5)	
H8A	0.3424 (19)	0.261 (6)	0.1652 (19)	0.042 (9)*	
H8B	0.302 (2)	0.500 (7)	0.1168 (19)	0.055 (10)*	
C9	0.05728 (14)	0.0322 (5)	−0.07822 (14)	0.0237 (5)	
C10	0.03648 (17)	0.2181 (5)	−0.13815 (16)	0.0341 (7)	
H10	0.0741	0.3502	−0.1371	0.041*	
C11	−0.04013 (18)	0.2079 (6)	−0.19945 (17)	0.0444 (8)	
H11	−0.0542	0.3339	−0.2399	0.053*	
C12	−0.09593 (17)	0.0147 (6)	−0.20168 (17)	0.0423 (8)	
H12	−0.1477	0.0092	−0.2435	0.051*	
C13	−0.07527 (17)	−0.1708 (6)	−0.14209 (18)	0.0417 (8)	
H13	−0.1131	−0.3025	−0.1432	0.050*	
C14	0.00090 (17)	−0.1621 (6)	−0.08100 (16)	0.0345 (6)	
H14A	0.0148	−0.2891	−0.0408	0.041*	
C15	0.13744 (15)	0.0420 (5)	−0.01207 (13)	0.0219 (5)	
C16	0.24976 (14)	0.1614 (5)	0.07732 (13)	0.0198 (5)	

### Atomic displacement parameters (Å^2^)

**Table:**

	*U*^11^	*U*^22^	*U*^33^	*U*^12^	*U*^13^	*U*^23^
N1	0.0273 (11)	0.0216 (10)	0.0205 (10)	−0.0031 (9)	0.0108 (9)	−0.0002 (8)
N2	0.0274 (11)	0.0216 (10)	0.0200 (10)	−0.0022 (9)	0.0099 (8)	−0.0016 (8)
N3	0.0306 (11)	0.0236 (11)	0.0193 (10)	−0.0060 (9)	0.0143 (9)	−0.0013 (9)
N4	0.0378 (13)	0.0325 (13)	0.0266 (12)	−0.0140 (11)	0.0182 (10)	−0.0090 (10)
C1	0.0229 (12)	0.0212 (12)	0.0217 (12)	0.0036 (10)	0.0082 (10)	−0.0010 (10)
C2	0.0293 (13)	0.0302 (14)	0.0244 (13)	−0.0007 (11)	0.0122 (11)	−0.0013 (11)
C3	0.0286 (14)	0.0343 (15)	0.0310 (14)	−0.0080 (12)	0.0079 (11)	−0.0104 (12)
C4	0.0268 (14)	0.0275 (14)	0.0419 (15)	−0.0067 (11)	0.0150 (12)	0.0017 (12)
C5	0.0476 (18)	0.0449 (17)	0.0391 (16)	−0.0131 (15)	0.0279 (14)	−0.0024 (14)
C6	0.0448 (16)	0.0356 (15)	0.0298 (14)	−0.0133 (13)	0.0197 (13)	−0.0075 (12)
C7	0.0249 (12)	0.0190 (12)	0.0176 (11)	0.0031 (10)	0.0085 (9)	0.0007 (9)
C8	0.0237 (12)	0.0228 (13)	0.0208 (12)	0.0009 (10)	0.0093 (10)	0.0011 (10)
N5	0.0246 (10)	0.0196 (10)	0.0214 (10)	0.0019 (8)	0.0072 (8)	−0.0015 (8)
N6	0.0264 (11)	0.0176 (11)	0.0250 (11)	0.0004 (9)	0.0067 (9)	0.0018 (9)
N7	0.0276 (11)	0.0219 (11)	0.0211 (10)	0.0015 (9)	0.0061 (9)	0.0004 (8)
N8	0.0267 (12)	0.0222 (12)	0.0223 (11)	0.0028 (9)	0.0037 (9)	−0.0001 (9)
C9	0.0223 (12)	0.0203 (12)	0.0277 (13)	0.0023 (10)	0.0091 (10)	−0.0052 (10)
C10	0.0383 (15)	0.0234 (13)	0.0338 (14)	0.0007 (12)	0.0073 (12)	0.0002 (11)
C11	0.0453 (18)	0.0347 (17)	0.0357 (16)	0.0085 (14)	−0.0022 (14)	0.0022 (13)
C12	0.0278 (15)	0.0420 (18)	0.0431 (17)	0.0073 (13)	−0.0008 (13)	−0.0139 (14)
C13	0.0285 (15)	0.0431 (17)	0.0506 (18)	−0.0101 (13)	0.0126 (13)	−0.0157 (15)
C14	0.0357 (15)	0.0313 (15)	0.0349 (15)	−0.0016 (12)	0.0123 (12)	−0.0024 (12)
C15	0.0261 (12)	0.0193 (12)	0.0218 (12)	0.0001 (10)	0.0113 (10)	−0.0022 (10)
C16	0.0241 (12)	0.0196 (12)	0.0166 (11)	0.0031 (10)	0.0092 (9)	−0.0009 (9)

### Geometric parameters (Å, °)

**Table:**

N1—C8	1.332 (3)	N5—C15	1.340 (3)
N1—C7	1.359 (3)	N5—C16	1.366 (3)
N2—C7	1.321 (3)	N6—C15	1.326 (3)
N2—N3	1.387 (3)	N6—N7	1.375 (3)
N3—C8	1.348 (3)	N6—H6N	0.87 (4)
N3—H3N	0.89 (3)	N7—C16	1.328 (3)
N4—C8	1.337 (3)	N8—C16	1.372 (3)
N4—H4A	0.92 (3)	N8—H8A	0.81 (3)
N4—H4B	0.85 (3)	N8—H8B	0.94 (4)
C1—C2	1.385 (3)	C9—C14	1.389 (4)
C1—C6	1.388 (3)	C9—C10	1.390 (4)
C1—C7	1.470 (3)	C9—C15	1.468 (3)
C2—C3	1.385 (4)	C10—C11	1.387 (4)
C2—H2	0.94	C10—H10	0.94
C3—C4	1.375 (4)	C11—C12	1.380 (4)
C3—H3	0.94	C11—H11	0.94
C4—C5	1.373 (4)	C12—C13	1.384 (4)
C4—H4	0.94	C12—H12	0.94
C5—C6	1.384 (4)	C13—C14	1.380 (4)
C5—H5	0.94	C13—H13	0.94
C6—H6	0.94	C14—H14A	0.94
			
C8—N1—C7	103.55 (19)	C15—N5—C16	102.86 (19)
C7—N2—N3	102.47 (18)	C15—N6—N7	110.6 (2)
C8—N3—N2	109.15 (19)	C15—N6—H6N	131 (2)
C8—N3—H3N	129.2 (18)	N7—N6—H6N	118 (2)
N2—N3—H3N	120.4 (18)	C16—N7—N6	101.93 (18)
C8—N4—H4A	118 (2)	C16—N8—H8A	115 (2)
C8—N4—H4B	121 (2)	C16—N8—H8B	113 (2)
H4A—N4—H4B	120 (3)	H8A—N8—H8B	119 (3)
C2—C1—C6	118.5 (2)	C14—C9—C10	119.3 (2)
C2—C1—C7	121.3 (2)	C14—C9—C15	120.1 (2)
C6—C1—C7	120.2 (2)	C10—C9—C15	120.7 (2)
C3—C2—C1	120.4 (2)	C11—C10—C9	119.7 (3)
C3—C2—H2	119.8	C11—C10—H10	120.2
C1—C2—H2	119.8	C9—C10—H10	120.2
C4—C3—C2	120.6 (2)	C12—C11—C10	120.7 (3)
C4—C3—H3	119.7	C12—C11—H11	119.6
C2—C3—H3	119.7	C10—C11—H11	119.6
C5—C4—C3	119.3 (2)	C11—C12—C13	119.7 (3)
C5—C4—H4	120.3	C11—C12—H12	120.2
C3—C4—H4	120.3	C13—C12—H12	120.2
C4—C5—C6	120.5 (3)	C14—C13—C12	119.9 (3)
C4—C5—H5	119.7	C14—C13—H13	120.0
C6—C5—H5	119.7	C12—C13—H13	120.0
C5—C6—C1	120.5 (3)	C13—C14—C9	120.7 (3)
C5—C6—H6	119.7	C13—C14—H14A	119.6
C1—C6—H6	119.7	C9—C14—H14A	119.6
N2—C7—N1	114.8 (2)	N6—C15—N5	110.0 (2)
N2—C7—C1	123.0 (2)	N6—C15—C9	123.7 (2)
N1—C7—C1	122.1 (2)	N5—C15—C9	126.3 (2)
N1—C8—N4	125.5 (2)	N7—C16—N5	114.6 (2)
N1—C8—N3	110.0 (2)	N7—C16—N8	122.9 (2)
N4—C8—N3	124.6 (2)	N5—C16—N8	122.4 (2)

### Hydrogen-bond geometry (Å, °)

**Table:**

*D*—H···*A*	*D*—H	H···*A*	*D*···*A*	*D*—H···*A*
N3—H3N···N2^i^	0.89 (3)	2.08 (3)	2.966 (3)	175 (3)
N4—H4A···N1^ii^	0.92 (3)	2.09 (3)	3.011 (3)	173 (3)
N4—H4B···N8^i^	0.85 (3)	2.25 (3)	3.091 (3)	170 (3)
N6—H6N···N5^iii^	0.87 (4)	2.04 (4)	2.879 (3)	159 (3)
N8—H8A···N2	0.81 (3)	2.41 (3)	3.206 (3)	168 (3)
N8—H8B···N7^iv^	0.94 (4)	2.19 (4)	3.115 (3)	169 (3)

Symmetry codes: (i) −*x*+1, *y*+1/2, −*z*+1/2; (ii) −*x*+1, −*y*+1, −*z*+1; (iii) *x*, *y*−1, *z*; (iv) *x*, *y*+1, *z*.
